# Multiple intracellular pathogen infections with ocular pathologies associated with adult-onset immunodeficiency due to anti-interferon-γ autoantibodies: a case report

**DOI:** 10.1186/s12879-024-09003-x

**Published:** 2024-01-12

**Authors:** Yan Ning, Qingliang Yu, Hanlin Liang, Siyao Wu, Siqiao Liang, Xiaona Liang, Zhiyi He

**Affiliations:** The First Affiliated Hospital of Guangxi Medical University, Nanning, Guangxi China

**Keywords:** Anti-interferon-γ autoantibodies, Nontuberculous mycobacteria, *Talaromyces marneffei*, *Mycobacterium tuberculosis*, Case report

## Abstract

**Background:**

Autoantibodies against interferon-γ (IFN-γ) can inhibit IFN-γ-dependent signal transducer and activator of transcription 1 phosphorylation and thus increase the risk of infection with intracellular pathogens, such as *Talaromyces marneffei* (TM), nontuberculous mycobacteria (NTMs), and *Mycobacterium tuberculosis* (TB). Here, we report a rare case of triple infection caused by TM, NTM, and TB in a human immunodeficiency virus–negative patient.

**Case presentation:**

A middle-aged female was admitted to our hospital after experiencing recurrent rash, cough, and expectoration for 4 months. She was successively diagnosed with NTM, TM, and TB infections without conventional immunosuppression-associated factors. However, after effective anti-infective treatment, the patient was confirmed to have allergic conjunctivitis and was successfully treated with corticosteroids and immunosuppressants. The most conspicuous characteristics were recurrent infection and immune disorders.

**Conclusions:**

High-titer anti-IFN-γ autoantibodies are strongly associated with severe and disseminated infections, such as NTM, TM, and TB. It is characterized by persistently high degree of inflammation and high immunoglobin levels.

## Background

Adult-onset immunodeficiency syndrome (AOIDS) due to anti-interferon-γ (anti-IFN-γ) autoantibodies is a distinct and emerging clinical entity and usually found in Southeast Asia [1–4]. It was first described in 2012 by Browne et al. [1, 5, 6]. Neutralizing anti-IFN-γ autoantibodies are detected in 88% of Asian adults with multiple opportunistic infections and associated with an adult-onset immunodeficiency akin to that of advanced human immunodeficiency virus (HIV) infection [1–3, 7]. Anti-IFN-γ autoantibodies (AIGAs) are considered susceptibility factors for infection by multiple intracellular pathogens, especially nontuberculous mycobacteria (NTM), *Talaromyces marneffei, Cryptococcus neoformans, Histoplasma capsulatum* [1, 2, 8]. In patients with high-titer AIGAs, the clinical presentations may vary by site of infection and related pathogens. The common clinical features of infected patients are multiple–lymph node enlargement, lung lesions, bone destruction, and skin lesions, and liver and spleen can be involved [2, 3]. Ocular pathologies caused by AIGAs are rarely reported. Here, we report a patient who had high-titer serum AIGAs and developed multiple infections by disseminated intracellular pathogens and ocular lesions. We then explore the underlying mechanism.

### Case presentation

A 61-year-old Chinese woman was admitted to our hospital on February 3, 2021 because of a 4 month history of cough and expectoration accompanied by multiple red rashes, edema, and painful subcutaneous nodules in the legs. She had been initially admitted and treated at a local hospital. Purified protein derivative (PPD) test result showed positive, but she was nonresponsive to piperacillin–tazobactam and hormone therapy. The vital signs during the initial examination were as follows: body temperature, 36 °C; blood pressure, 112/72 mmHg; heart rate, 106 beats/min; and respiratory rate: 20 breaths/min. Physical examination revealed painful subcutaneous nodules in the left calf. The patient had no previous history of immunodeficiency or exposure to immunosuppressants.

**Fig. 1:**
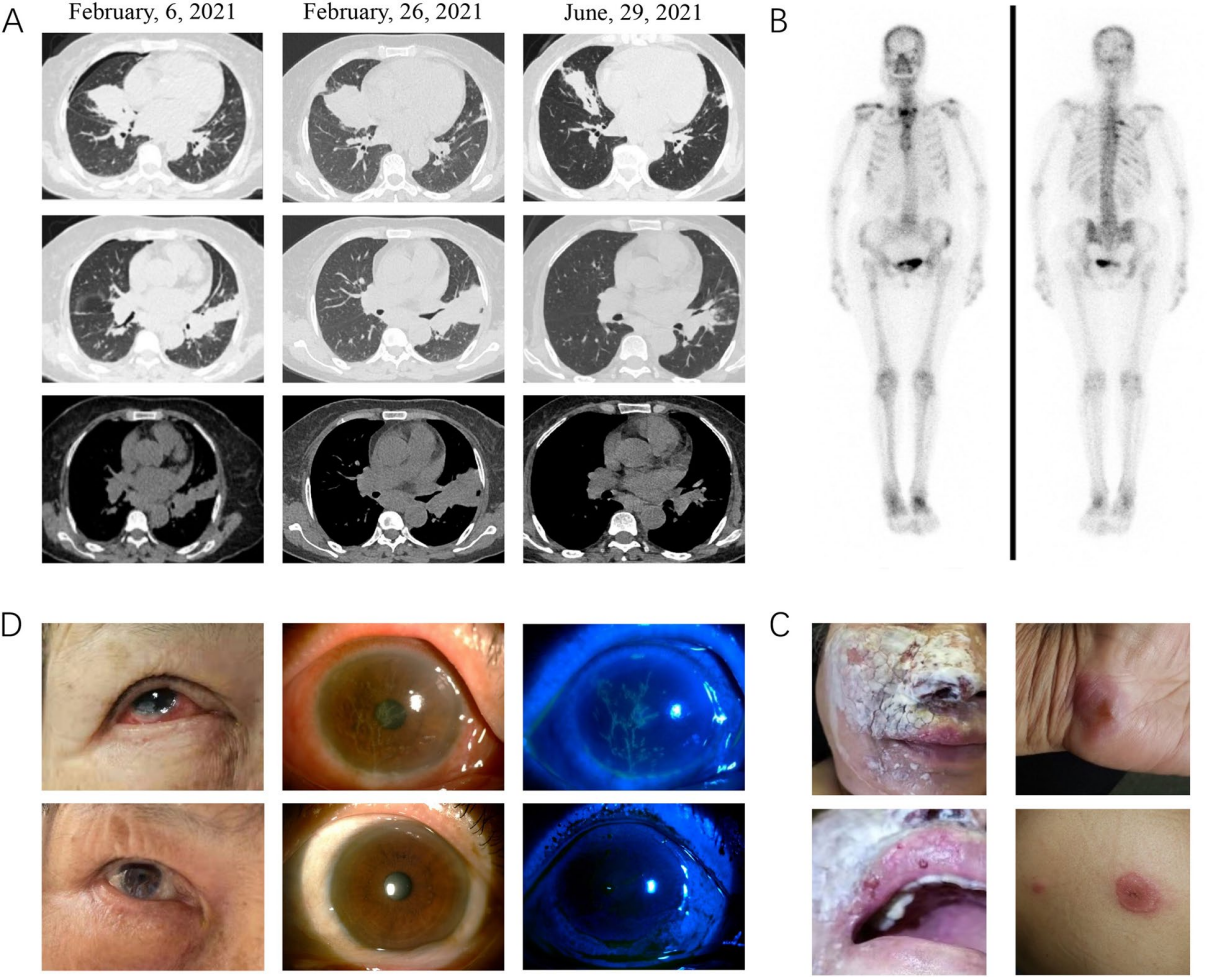
**A** Computed tomography dynamic monitoring series: pulmonary lesions (the middle lobe of the right lung and the upper lobe of the left lung), lymphadenopathy (hilus and mediastinum), worsening of lung lesions before antifungal therapy, and obvious absorption with regular antifungal and antibacterial regimen use. **B** Emission computed tomography: significantly increased uptake in multiple bones including skull, sternum, multiple ribs, left iliac bone, right forearm and femur, and bilateral ankle joints. **C** Multiple skin lesions (submental and right submandibular skin, hands, and back) **D** Ocular lesions, hyperemia, and edema of right eye and the corneal fluorescein staining showed pseudodendritic lesions before and after glucocorticoid and immunosuppressant use, showing dramatic improvement

Initial laboratory examinations indicated that elevated levels of white blood cells (WBCs) and C-reactive protein (CRP) and increased erythrocyte sedimentation rate (ESR) and immunoglobulin E (IgE) level (Table 1). Notably, other routine biochemistry, kidney, and liver function tests yielded normal findings. Lymphocyte subset counts and percentages were normal. HIV serology and reverse transcription polymerase chain reaction for COVID-19 were negative. Chest computerized tomography (CT) revealed bilateral pulmonary consolidation with hilar and mediastinal lymphadenopathy (Fig. 1A). Metagenomic next-generation sequencing (mNGS) of the bronchoalveolar lavage fluid (BALF) was negative. The biopsied tissue from the skin of her left leg showed chronic suppurative inflammation. *M. tuberculosis* was identified using matrix-assisted laser desorption–ionization time of flight mass spectrometry (MALDI–TOF MS) from biopsied tissues. Despite the lack of evidence of NTM infection, we did not rule out NTM infection because the patient presented with skin and lung lesions and rapid disease progression. Therefore, empirical anti-NTM treatments with cefoxitin, moxifloxacin, azithromycin, anti-tuberculosis rifampicin, and ethambutol regimens were successively administered, but no improvement was observed.

**Table 1:** Laboratory data, pathogen, and diagnostic method during previous hospitalization

Time	Laboratory data				Pathogen	Diagnostic method
	WBC (× 10^9^/L)	CRP (g/L)	ESR (mm/h)	IgE (IU/mL)		
2021.2.3–2021.2.9	13.44	136.9	82	185.4	*M. tuberculosis*	MALDI-TOF MS of skin tissue
2021.2.25–2021.3.12	12.44	159.65	126	202.5	*M. intracellulare*	mNGS of BALF
2021.4.23–2021.5.21	9.12	47.67	108	213.1	*M. tuberculosis*; *M. tuberculosis*	Culture and MALDI-TOF MS of lymph node tissue

*mNGS* Metagenomics next-generation sequencing detection, *BALF* Bronchoalveolar lavage fluid, *MALDI-TOF MS* Matrix-assisted laser desorption/ionization-time of flight mass spectrometry

By the end of February 2021, the patient was hospitalized twice for recurrent cough and expectoration. Meanwhile, she presented with swelling and pain of the bilateral calf and started experiencing fever. The maximum body temperature was 39.5 °C. Chest CT displayed progressed consolidation in the bilateral lungs (Fig. 1A). We sent BALF for mNGS again, and *Mycobacterium intracellulare* was detected. The patient continued antibacterial therapy (comprising rifampicin, ethambutol, moxifloxacin, and azithromycin). However, the patient was hospitalized again because skin lesions (including the submental and right submandibular skin, hands, and back) increased, and redness in her right eye was observed during 2 months of follow-up (Fig. 1C). Thus, moxifloxacin was stopped, and isoniazid was added to the treatment regimen. Ultrasound showed bilateral cervical lymphadenopathy, and the re-examination of chest CT indicated that the lung lesions were slightly absorbed (Fig. 1A). The emission CT showed a significantly increased uptake in multiple bones, including the skull, sternum, multiple ribs, left iliac bone, right forearm and femur, and bilateral ankle joints (Fig. 1B). To obtain definitive pathogen evidence, we performed MALDI-TOF MS, and pathogens from the right cervical lymph node were cultured. *M. tuberculosis* was detected by MALDI-TOF MS, and *T. marneffei* was cultured from the biopsied lymph node. Amphotericin B was added as antifungal therapy, and the antibacterial regimen was simultaneously adjusted to rifampicin, ethambutol, isoniazid, and levofloxacin because of an adverse reaction of the gastrointestinal tract to azithromycin. The patient was anti-IFN-γ autoantibody positive with a titer of 1:2500, as determined by enzyme-linked immunosorbent assay. The patient was diagnosed as positive to IFN-γ autoantibodies with disseminated *T. marneffei*, NTM, and *M. tuberculosis* infections.

After nearly 6 months of anti-infective treatment, the patient’s clinical condition remained stable, skin symptoms improved, and lung lesions were absorbed. However, she complained of amaurosis, redness, photophobia, and tearing of right eye with obvious fatigue (Fig. 1D). The patient’s WBC count was normal, but immunological and inflammatory tests revealed elevated CRP (47.64 mg/L), ESR (123 mm/h), and IgE concentration (269.8 g/L; Table 1). The ophthalmic clinic examination revealed conjunctivitis, hyperemia, and edema of the right eye, and the corneal fluorescein staining showed pseudodendritic lesions. We suspected that the root cause was immune-mediated allergic conjunctivitis caused by anti-IFN-γ autoantibodies. This condition followed the patterns of types I and IV hypersensitivity mechanisms. Corticosteroid treatment (methylprednisolone 16 mg every 24 h) and local application of tacrolimus eye drops were initiated. After 2 weeks, the ocular symptoms improved dramatically, and inflammation, immunoglobulin level, and the titer of anti-IFN-γ antibody were reduced (Fig. 1D). Thereafter, the patient’s clinical condition remained stable without relapse.

## Discussion and conclusions

AOIDS usually presents as chronic, recurrent, and hard-to-control infections or unusual serious infections that can be effectively treated with aggressive antibiotic therapy [9]. Skin manifestation is a frequent feature of the syndrome, which includes infections of the skin and reactive conditions, such as *Sweet* syndrome, pustular eruption, and panniculitis [2, 8]. Multiple-organ involvement is another feature of AOIDS. The lungs are the most affected, followed by the lymph nodes, skin, bones, joints, liver, and spleen [2, 3]. AOIDS due to high-titer of AIGA is the most common underlying immunodeficiency in HIV-negative patients [3, 4, 6, 9]. In China, anti–IFN-γ autoantibodies in HIV-negative patients with *T. marneffei* infections are primarily distributed in southern regions, such as Guangdong and Guangxi [3, 4]. Co-infection by *M.tuberculosis*, NTM, and *T. marneffei* is extremely rare. Our patient had no previous underlying diseases, such as autoimmune diseases, hematological malignancies, tumors, or diabetes. The patient was from Guangxi and resided there all her life. HIV-negative hosts, especially those infected by *T. marneffei* with or without other opportunistic infections, develop intracellular opportunistic infections. Thus, clinicians should be vigilant for immunodeficiency due to AIGAs. IFN-γ plays a key role in activating phagocytes to clear engulfed pathogens in humans. AIGAs may inhibit the CD4 T cells’ IFN-γ/pSTAT-1/Th1 pathway, ultimately leading to a severely compromised Th1 response [4, 5, 10]. Thus, the risk of infection by severe and fatal multiple intracellular pathogens increases [5, 10]. AOIDS is highly associated with two specific HLA class II alleles: HLA-DRB1*16:02/DQB1*05:02 and HLA-DRB1*15:02/DQB1*05:01 [3, 4, 9]. Unfortunately, we did not conduct AIGAs on uninfected family members, and no unifying genetic theory was found for this patient. The detailed mechanism by which IFN-γ contributes to the control of *T. marneffei* and NTM in vivo remains to be determined.

In addition, immune disorders caused by AIGAs play an important role. The ocular symptoms were associated with intracellular pathogens infections. We observed that the patient had ocular symptom during her third hospitalization. As the infection was not controlled, the patient’s ocular symptom was first considered to have been caused by pathogen infection according to the clinical characteristics and auxiliary examinations. After anti-infective treatment, the patient’s clinical condition remained stable. Ocular symptoms appeared with increased immunoglobulin levels (IgE and IgG) and anti-IFN-γ titers. Allergic conjunctivitis may have been mediated by AIGAs, given the absence of evidence of infection. And Corticosteroid and immunosuppressant therapeutic response also support this suspect. Persistent elevated inflammation and immunoglobin level are the conspicuous characteristics. However, reports about how AIGAs cause systemic autoimmunity are rare.

Treatments for AIGA-related AOIDS target the complications of infection or the auto-antibodies themselves [11, 12]. To date, no standardized method to treat patients with adult-onset immunodeficiency with anti-IFN-γ autoantibodies has been established, except anti-infective treatment. Rituximab, exogenous IFN-c, plasmapheresis, and cyclophosphamide have been used to treat refractory infections [4, 11–14]. Corticosteroids and anti-IFN-γ autoantibodies have not been explored. Nevertheless, our patient benefited from corticosteroids. When an infection is not controlled, corticosteroids may aggravate patients' conditions. Therefore, attention should be paid to balancing the benefits of corticosteroid treatment and infection risk, and the timing and dose of corticosteroid treatment need further attention.

In summary, we reported a case of an HIV-negative woman with AIGA who developed multiple disseminated intracellular organism infection and allergic conjunctivitis. Her ocular symptoms may be related to elevated immunoglobulin levels (IgE and IgG) and anti-IFN-γ titers. Long-term anti-infective and corticosteroid treatment improved clinical manifestations.

## Data Availability

The study’s datasets can be obtained from the corresponding author upon reasonable request.

## Abbreviations

IFN-γ: Interferon-gamma
NTM: Nontuberculous mycobacteria
TM: *Talaromyces marneffei*
TB: *Mycobacterium tuberculosis*
WBC: White blood cell
ESR: Erythrocyte sedimentation rate
CRP: C-reactive protein
CT: Computerized tomography
BALF: Bronchoalveolar lavage fluid
ELISA: Enzyme-linked immunosorbent assay
